# The role of semantics, pre-emption and skew in linguistic distributions: the case of the *un*-construction

**DOI:** 10.3389/fpsyg.2013.00989

**Published:** 2013-12-25

**Authors:** Paul Ibbotson

**Affiliations:** Centre for Childhood, Learning and Development, Open UniversityMilton Keynes, UK

**Keywords:** generalizations, verbal semantics, pre-emption, skew, diachronic use

## Abstract

We use the Google Ngram database, a corpus of 5,195,769 digitized books containing ~4% of all books ever published, to test three ideas that are hypothesized to account for linguistic generalizations: verbal semantics, pre-emption and skew. Using 828,813 tokens of *un*-forms as a test case for these mechanisms, we found verbal semantics was a good predictor of the frequency of *un*-forms in the English language over the past 200 years—both in terms of how the frequency changed over time and their frequency rank. We did not find strong evidence for the direct competition of *un*-forms and their top pre-emptors, however the skew of the *un*-construction competitors was inversely correlated with the acceptability of the *un*-form. We suggest a cognitive explanation for this, namely, that the more the set of relevant pre-emptors is skewed then the more easily it is retrieved from memory. This suggests that it is not just the frequency of pre-emptive forms that must be taken into account when trying to explain usage patterns but their skew as well.

## Introduction

People are both creative and conventional with their language use. Linguistic theory must therefore perform something of a balancing act: granting people enough inventiveness to generate new ways of speaking while at the same time assuming they conform with the linguistic norms of their community. Describing the cognitive and social processes that give rise to this situation is one of the central goals of linguistics.

To take an example, words beginning with the prefix *un*- such as *unlock* and *unbutton* typically designate reversible actions. It sounds somewhat odd, however, to say ?*unclose a door* or ?*unlift one's arms* even though these are reversible actions and semantically interpretable statements. Here we make use of a relatively new tool to investigate some of the cognitive processes that might be involved in restricting the *un*- generalization. In 2011 Google made public its Ngram database, a corpus of 5,195,769 digitized books containing ~4% of all books ever published. Using the *un*-construction as a test case, we use the Ngram database to test three ideas that are hypothesized to account for linguistic generalizations: verbal semantics, pre-emption and skew.

In construction grammar the form of constructions carry meaning independent of the meaning of the items that appear in those constructions. For example, *the gazzer mibbed the toma* is typically interpreted as a transitive not because of the compositionality of the meaning of the items (they are meaningless) but by analogy to other sentences of the form *the X Yed the Z*. Because this approach allows constructional form to carry meaning as well as lexical items, items can be more or less semantically compatible with the construction they appear in. The prediction is that that the more compatible an item's semantics is with that of the construction the more likely it is to appear in that construction. Semantic fit has thus been suggested as a possible mechanism that can explain linguistic patterns, such as why certain forms resist generalizations in certain directions (e.g., Pinker, 1989; Ambridge et al., 2011). With regard to the semantics of the un-construction, Whorf (1956) suggested it could be described as a kind of cryptotype; a cluster of features implicit to the language user (hence crypto), that tend to co-occur—no single one of which could be considered the definitive meaning of the un-form but taken together describe the semantic core of un prefixation. These features include whether one object affects another, touches it, and distorts it for example. The semantic fit hypothesis predicts that un-forms with features that are the closest match to the un-form cryptotype will be the ones that most readily appear in the un- construction. Those forms with semantic features at odds with the semantics of the construction will be resisted and therefore rare in the language.

A further explanation to the distribution of un-forms is that the process of hearing a verb with sufficient frequency in a particular grammatical context prevents that verb from appearing in other structures unless it is also attested in those structures (Braine and Brooks, 1995; Brooks and Tomasello, 1999). The idea that speakers are conservative about their generalizations with items that are entrenched in the contexts they hear them has received some empirical support (e.g., Brooks et al., 1999; Theakston, 2004; Ambridge et al., 2008). This partly answers why some forms resist being “exported” out the construction they are entrenched in, however, it does not explain why it is these forms that show entrenchment in the first place, rather than some other (semantically possible) forms. One answer to this could simply be linguistic drift. Historically-speaking, some forms have randomly walked into a position where they are more frequent in the language and through the principle of least effort it is these forms that remain entrenched. Despite this, we still need a mechanism that explains, where innovations could occur, and are semantically plausible, why these still might be resisted. One suggestion is that as well as items showing resistance to being “exported” out of their usual grammatical context (entrenchment) there is an additional resistance to them being “imported” into a new context if that linguistic niche is already filled with a successful competitor. Thus, if a verb is repeatedly used in a construction, (e.g., *I filled the cup with water*) that serves the same communicative function as a possible unattested generalization (e.g., *I filled water into the cup)*, then the attested form blocks or pre-empts the possible generalization (Pinker, 1981; Clark, 1987; Goldberg, 1995).

In previous work, Ambridge (2013) found experimental support that adults and children are sensitive to both the semantic fit and pre-emption when judging the acceptability of various *un*-forms. In his study children aged 5–6, 9–10, and adults rated the acceptability of *un*- prefixed forms of 48 verbs (and, as a control, bare forms). The higher the frequency of potentially pre-empting forms (e.g., *open*), the less willing participants were to accept a particular un- form (e.g., ^*^unclose). Semantic fit between un-forms and the un-construction was also a significant positive predictor of acceptability, for all age groups. The focus of that study was synchronic use; investigating the mechanisms by which children retreat from overgeneralization in the process of language acquisition. Thus, semantic features and pre-emption were originally concepts developed to solve problems in the language acquisition domain. Here we apply these processes in the diachronic domain; building on Ambridge's findings we asses to what extent semantic fit, pre-emption and skew (which Ambridge did not consider) can explain the historical distribution and frequency of un-forms.

Skewed distributions have been shown to have a beneficial role in learning linguistic and non-linguistic categories [Elio and Anderson, 1984; Avrahami et al., 1997; Gentner et al., 2002; Casenhiser and Goldberg, 2005; Year, 2009, but see Cordes and Krajewski, in preparation; Year and Gordon, 2009,^1^]. Cognitive anchoring is one mechanism that has been suggested to play a role in this effect (e.g., Goldberg et al., 2004; Goldberg, 2006, p. 88). According to the theory, a high frequency exemplar that emerges early in learning provides a salient standard of comparison against which subsequent instances are judged. For example, Casenhiser and Goldberg (2005) investigated how children map a novel meaning onto a new phrasal form, and how this process could be facilitated by constructions that are centered around prototypical verbs. Fifty one 5- to 7-year-old children watched a short set of video clips depicting objects appearing in various ways. Each scene was described using a novel verb embedded in a novel construction. The novel construction NP1 NP2 Novel Verb + O was indicative of the meaning of “appearance.” The different verbs indicated manner of appearance. For example, children heard *the spot the king moopo-es*, they then saw a video-clip where a spot appears on the king's nose, and then they *heard the spot the king moopo-ed*. There were five novel verbs and 16 examples of the construction so that in the more balanced condition the proportion of verb types were arranged as: 4-4-4-2-2 and in the skewed frequency condition they were arranged as: 8-2-2-2-2.

Children who watched the videos and heard the accompanying description were able to match new descriptions that used the novel construction with new scenes of appearance. There was a facilitative effect for the disproportionately high frequency of occurrence of a single verb in a particular construction such as has been found to exist in naturalistic input to children (Goldberg et al., 2004, p. 3). The statistical skew in the input on this view facilitates relatively rapid category construction compared to flatter distributions because a minority of high frequency types provide a relevant anchor that serves to organize memory and reasoning about other types.

The idea here is that a similar process may be at work in restricting *un*-construction generalizations. The more the set of relevant pre-emptors is skewed then the more easily an alternative form for the un-construction is retrieved from memory because it has a higher resting activation level. For example, *unclose* might be pre-empted as a generalization not just because *open* is frequent [as established by Ambridge (2013)] but because *open* is a clear modal alternative to *unclose* in the set of forms competing to express a similar concept, making it relatively easier to access from memory compared with flatter distributions.

To add a diachronic perspective to this analysis we make use of so-called “Big Data” in the form of Google's Ngram copus. Using this dataset Michel et al. (2011) showed how it was possible to provide insights about fields as diverse as lexicography, the evolution of grammar, collective memory, the adoption of technology, the pursuit of fame, censorship, and historical epidemiology.

A corpus of words extracted from written text is clearly different from that of spoken language. In general the printed word is subject to more deliberation both on behalf of the writer and the publisher (it is more planned, proofed, edited and so on). One might expect more conservative use of forms on this basis. However, the written word does not rule out non-conventional use. In creative writing genres such as fiction and poetry we might expect more unconventional use than in spoken discourse where authors push and play with what is considered conventional (many of these texts will of course be quoting dialog also). Second, the relative performance of Ngram to other printed word corpora has proved particularly useful for detecting linguistic “dark matter.” Using Google Ngram Michel et al. (2011) estimated that 52% of the English lexicon—the majority of the words used in English books—consists of lexical material undocumented in standard references like the *Oxford English Dictionary* and the *Merriam-Webster Unabridged Dictionary*. If unconventional forms (by today's standards), like *unwent*, *uncame*, and *ungive*, were once more frequent, the increased coverage of this corpus gives us a better chance of detecting them. Third, assuming written text is relatively impoverished in unconventional un-forms, if there are enough raw forms to perform the analysis, we are looking at changes in use within the bounds of conservative use and not making claims about absolute levels (which may or may not be higher in spoken language).

Here we focus on the use of *un*-forms and their respective preemptors from 1800-2008. We assess the extent to which the combination of *semantics*, *pre-emption* and *skew* can account for usage patterns of the *un*-construction.

## Methodology

### Semantic feature rating for un-forms

In order to see the effect of semantics on historical frequency we need some measure of the extent to which particular verbs exhibit the properties of the un-form semantic cryptotype. Li and MacWhinney (1996) asked 15 native English speakers to rate un verb forms such as *unfroze*, *unbuckled* and *unbent* on the basis of whether they exemplified the 20 features thought to be relevant from Whorf's earlier cryptotype work (Whorf, 1956):

(1) Mental activity, (2) Manipulative action, (3) Circular movement, (4) Change of location, (5) Change of state, (6) Resultative, (7) A affects B, (8) A touches B, (9) A distorts B, (10) A contains B, (11) A hinders B,(12) A obscures B, (13) A surrounds B, (14) A tightly fits into B, (15) A is a salient part of B, (16) A and B are separable, (17) A and B are connectable, (18) A and B are interrelated, (19) A and B are in orderly structure, (20) A and B form a collection.

Thus, each verb had a score of between 0 and 15 for each semantic feature, corresponding to the number of participants who judged the feature to be relevant to the verb's meaning. Using a subset of Li and MacWhinney's original verb list, Ambridge (2013) condensed the 20 semantic predictors using Principle Components Analysis, selecting the single component with the largest eigenvalue. The result of this is a single value for each verb showing how well (or poorly) it fits the cluster of semantic features of the un-prefixation cryptotype. We use this value to cluster our verb types in our historical analysis and to analyze the verb's relationship to the skew of its pre-emptor set (discussed later). For example, *unchained* scores highly on many of the semantic features (and has a value of 1.92), *unopened* less so (−0.59) and *unasked* is a poor match for the semantic features (−1.46). The full list of verbs used here and their associated semantic values, from worst to best, is displayed below.

Unstood (−1.49807), unasked (−1.46745), unembarrassed (−1.37024), unallowed (−1.2649), unbelieved (−0.91602), unbent (−0.83763), undeleted (−0.82364), unreeled (−0.77099), uncrumpled (−0.70693), untightened (−0.69449), unstraightened (−0.69449), unopened (−0.59181), unpressed (−0.29138), unfroze (−0.10969), unmasked (−0.04196), unveiled (0.020561), unhooked (0.267467), unloosened (0.282053), unfilled (0.520944), unzipped (0.613764), unbuttoned (0.676796), unlaced (0.713534), unleashed (0.822933), uncorked (1.003491), unbandaged (1.078561), unfastened (1.097466), unlocked (1.396762), unlatched (1.536216), unbuckled (1.594093), unchained (1.927338).

### Pre-emptors

We use all the *un*-form pre-emptors collected as part of Ambridge (2013; personal correspondence). For each bare stem verb (e.g., *chain, bandage, freeze*) Ambridge asked 15 adult participants to do the following:

think up as many words as you can (maximum = 5) that mean the reversal of this action. Sometimes, there may be no suitable word, but always try as hard as you can to come up with at least one, even if it is not precisely the right meaning. However, you should NOT write words that you would consider “ungrammatical” (i.e., not real English words). VERY IMPORTANT: You MAY NEVER write an *un*- word, even if this word has the right meaning. For example, if the action is bolt, then *unbolt* would have the right meaning BUT YOU MAY NOT WRITE UNBOLT. Instead, you must try to come up with alternatives that do NOT start with *un*-.

**Figure 1:**
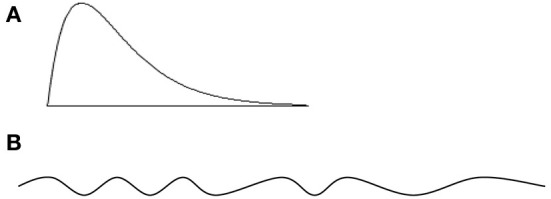
**(A)** Ditransitive distribution. Ditransitive {give, give, give, give, read, pass}. **(B)** Transitive distribution. Transitive {get, have, want, take, find, put, do, eat, play, see, like, say…}. Figure from Ibbotson and Tomasello (2009).

Ambridge chose the two most commonly suggested pre-empting forms for each verb, for example for *unsqueeze* it was *release* and *loosen*. For each pair of pre-empting forms, Ambridge calculated the natural log of *N* + 1, where N was the frequency of the pre-emptor in the British National Corpus. These two values were then summed to yield the pre-emption measure. Ambridge also calculated various other measures (e.g., frequency of the single most commonly suggested form, sum frequency of the top two forms/all forms, number of suggested forms, number of participants suggesting the top form), but all were less successful as predictors of *un*- form acceptability. As Ambridge notes, the advantage of including only the two most commonly suggested forms—as opposed to all forms—is that it excludes the more marginal examples that were only suggested by one or two participants. However, we consider all pre-emptive forms here that were suggested by at least one participant in Ambridge (2013) as we are not only interested in the top two pre-emptive forms but the shape of the distribution of the forms (analysis 3). The number of types and tokens for a set of pre-emptors associated with each verb was recorded and the skew calculated using the adjusted Fisher-Pearson standardized moment coefficient, a common statistical measure of skew defined by Equation (1).

(1) $$S=\frac{n}{\left(n-1\right)\left(n-2\right)}\sum {\left(\frac{x_{j}-\bar{x}}{s}\right)}^{3}$$

where *n* is the set of numbers for which we want to calculate skew, where *x_j_* is any particular score within that set, and *s* is the estimated standard deviation. A potential concern with this statistical measure is that is it does not completely capture the sense in which skew has been discussed in the linguistic literature. Recall, Casenhiser and Goldberg (2005) used a skewed distribution in their experiment of 8-2-2-2-2 with one item (represented by the 8 here) making up the lion's share of the token count in comparison with a flatter distribution of 4-4-2-2-2-2. The analogous example in English is where the verb *give* makes up the lion's share of ditransitive uses whereas the transitive construction has a flatter distribution with no one item being the clear modal verb (Figures 1A,B).

Returning to the present measure, the Fisher-Pearson skew defines any distribution that is symmetrical about the mean (e.g., [5,5,5], [3,4,5], [1,5,9]) as equally non-skewed and returns a value of 0—based on the fact that the mean and median are the same for these distributions. Distributions count as skewed when they are not symmetrical about their means (e.g., [1,5,13]). Now, a distribution of [1,5,9] would count as skewed under most linguistic discussions of skew (Goldberg's definition of *give* in the ditransitive for example; Goldberg et al., 2004) so in theory there is potentially a disconnect between the cognitive a anchoring definition of skew and our definition. However, all of our samples showed some degree of skew due to the very robust finding that natural language follows something like a power-law distribution (Zipf, 1935/1965). So in practice, we were only ever measuring the degree to which the sample is skewed, and given that there are many more ways to be non-symmetrically distributed about the mean than to be symmetrically distributed this problem was avoided. None of the preemptor sets analyzed in this study were symmetrically distributed about the mean (which would be confounded with a flat distribution) so we could be confident that the measure was capturing the degree to a sample was skewed. For example, for the possible un-prefixation *unstraighten* the pre-emptor set was as follows [12-2-2-1-1-1…] which in terms of types was respectively *bend*-*crease*-*curl*-*curve*-*constrict*-*misalign*… While the examples have been chosen here from opposite ends of the flat/skewed spectrum to illustrate a point, the measure of skew employed here is inherently continuous and so can capture the extent to which a distribution is skewed, rather than some binary distinction between flat and skewed.

### Corpus extraction procedure

We extracted the same set of 48 verbs Ambridge used in order to have independent semantic and pre-emption measures. We obtained the time series of word frequencies via Google's Ngram tool (http://books.google.com/ngrams/datasets) in the 1-gram English dataset (combining both British and American English). The corpus gives information on how many times, in a given year, a 1-gram or an n-gram is used, where a 1-gram is a string of characters uninterrupted by space. For each stemmed word we collected the occurrences (case insensitive) in each year from 1800 to 2008 (inclusive). Because the number of books scanned in the data set varies from year to year, we normalized the yearly amount of occurrences using the occurrences, of the word “*the*” for each year, which is considered as a reliable indicator of the total number of words in the data set. We normalized by the word “*the*,” rather than by the total number of words, to avoid the effect of the influx of data, special characters, and so on.

As Ambridge notes “some verbs that are ungrammatical in verbal *un*-prefixed form (e.g., ^*^Bart unembarrassed everyone) may appear in an adjectival past-participle *un*- form with the general meaning of “not,” with no reversal implied (e.g., a person may be described as unembarrassed, meaning simply “not embarrassed”)” (Ambridge, 2013, p. 36). For that reason, we restricted our extraction to verbal forms, made possible because the Ngram corpus is tagged for basic syntactic categories.

## Analysis and discussion

**Figure 2:**
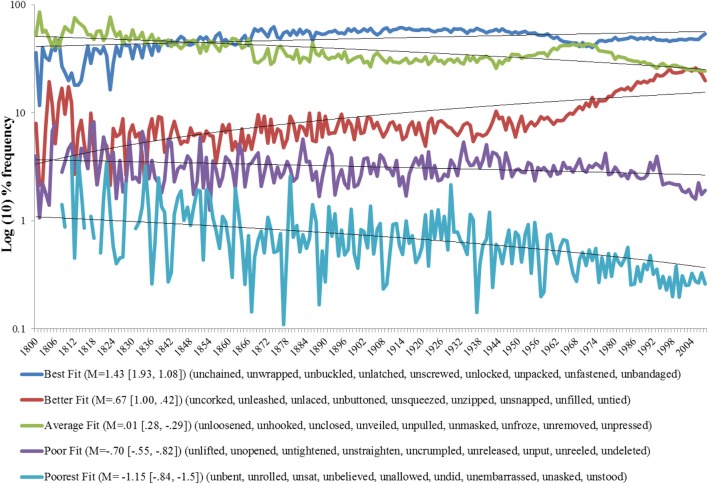
**Historical trends of un-forms (*N* = 828, 813) as a function of semantic rating.** M = mean semantic rating for the nine verbs in each group, ranges are in square parentheses, verbs in curly parentheses. Note the y-axis is relative frequency, thus at any slice on the x-axis values sum to 100%. We are interested in whether the historical proportion of un-forms forms is explainable in terms of their semantic rating. Linear lines of best fit are plotted to support the Pearson correlation (see analysis below).

First we focus on the semantics of the 828,813 tokens of *un*-forms extracted from the corpus. *Unwent*, *uncame*, and *ungave* which were included in Ambridge (2013) returned zero hits from the corpus and so are not included in the subsequent analysis. To get an overall impression of how the semantic character of the *un*-construction has changed relative to present-day judgments, each verb was assigned a semantic rating as calculated by Ambridge (see Methods). We then ranked these verbs from highest to lowest semantic rating and divided them into five groups of 9 verbs. Thus, the five groups represent better to worse examples of the *un*-construction cryptotype as judged by the semantic standards of present-day raters, and are labeled “best fit,” “better fit,” “average fit,” “poor fit,” and “poorest fit.” The result of this is a historical frequency plot of the *un*-form as a function of *un*-semantics, Figure 2.

To examine whether there has been any significant change over time, for each semantic group we conducted a Pearson bivariate correlation between the time (in years) and the corpus frequency Table 1.

**Table 1:** **Pearson correlation between semantic rating and year**.

**Semantic rating**	**%**	**Raw**
Best fit	*r*_(209)_ = 0.461, *p* < 0.001	*r*_(209)_ = 0.558, *p* < 0.001
Better fit	*r*_(209)_ = 0.645, *p* < 0.001	*r*_(209)_ = 0.639, *p* < 0.001
Average fit	*r*_(209)_ = –0.714, *p* < 0.001	*r*_(209)_ = –0.307, *p* < 0.001
Poor fit	*r*_(209)_ = –0.254, *p* < 0.001	*r*_(209)_ = 0.03, *p* = 0.667
Poorest fit	*r*_(209)_ = –0.333, *p* < 0.001	*r*_(209)_ = –0.270, *p* < 0.001

**Figure A1:**
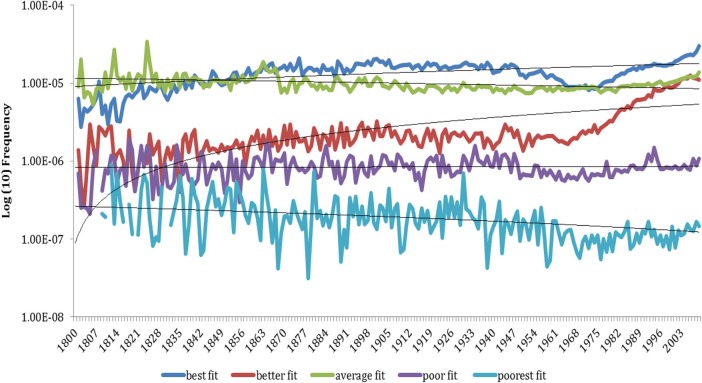
**Historical trends of un-forms (*N* = 828, 813) as a function of semantic rating, raw frequency**.

**Figure A2:**
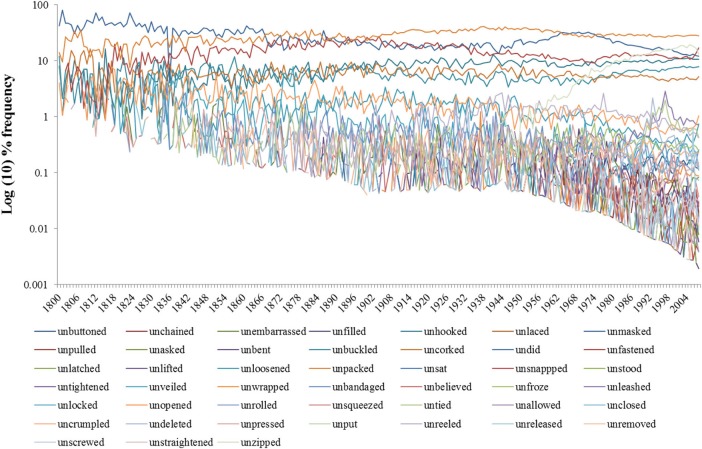
**Historical trends of un-form items**.

**Figure A3:**
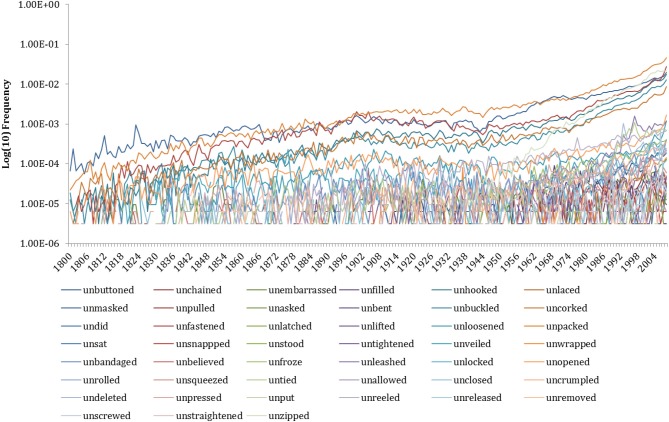
**Historical trends of un-forms (*N* = 828, 813) item analysis on raw frequency**.

The results show an increasing trend (significant positive correlations) in the frequency of the best and the better fit semantic categories. The data also show a decreasing trend for average, poor and poorest fit semantic categories. On the advice of one reviewer we also analyzed the historical trends based on the raw frequency data (raw column Table 1 and Figure A1 in appendix; For those interested, item analyses are also presented in the Appendix, both for proportions, Figure A2, and raw frequencies, Figure A3). The pattern observed in the proportion analysis is largely confirmed in the raw frequency analysis. The two analyses answer slightly different questions (i) can semantics predictive the frequency of un-forms relative to other un-forms and (ii) whether semantics predicts the relative frequency of un-forms as a function of corpus frequency (in this latter analysis we divide raw scores by the frequency of “the” as outlined in the procedure). What this means is that the semantic crypotype identified and tested by Whorf (1956), Li and MacWhinney (1996), and Ambridge (2013) is a good predictor of how frequency of these forms changes over time, namely, those with the best fit have been getting more frequent those with the poorer fit have been getting less frequent. This does not tell us whether the rank order of the semantic fit categories is what we would expect from their semantics. To examine this we compared the rank order of the semantic groups against what we would expect, namely those with a better fit to the cryptotype should be more frequent (and thus a higher rank) than those with a poorer fit, Table 2.

**Table 2:** **Rank order of frequency of different groups as a function of year (25 year windows)**.

	**Predicted rank**	**1800–1825**	**1826–1850**	**1851–1875**	**1876–1899**	**1900–1925**	**1926–1950**	**1951–1975**	**1976–1999**	**1999–2008**
Best fit	1	2	2	1	1	1	1	1	1	1
Better fit	2	3	3	3	3	3	3	3	3	3
Average fit	3	1	1	2	2	2	2	2	2	2
Poor fit	4	4	4	4	4	4	4	4	4	4
Poorest fit	5	5	5	5	5	5	5	5	5	5
		Rho(*N* = 5)	Rho(*N* = 5)	Rho(*N* = 5)	Rho(*N* = 5)	Rho(*N* = 5)	Rho(*N* = 5)	Rho(*N* = 5)	Rho(*N* = 5)	Rho(*N* = 5)
		0.7, *p* = 0.188	0.7, *p* = 0.188	0.9, *p* = 0.037	0.9, *p* = 0.037	0.9, *p* = 0.037	0.9, *p* = 0.037	0.9, *p* = 0.037	0.9, *p* = 0.037	0.9, *p* = 0.037

The results show the semantic rating does a good job overall of predicting the rank order of the frequencies over the historical period of English use covered by Google Ngram. From 1800 to 1850 it predicts the rank order of the poor and poorest fit categories but fails to predict the correct rank of any other categories. From 1851 to 2008 the predicted rank is a statistically significant predictor of the actual rank. The crypotype measure predicts the rank frequency of the best, poor and poorest groups, and predicts the better fit should be average fit and vice versa (given the historical trend observed in Figure 2, this might well be corrected in the near future as the frequency trends cross each other).

Using the British National Corpus Ambridge (2013) failed to find any such correlation between semantic rating and frequency of use. This is a little surprising as studies of this kind usually report a positive correlation between the extent to which items are rated as a good examples of their kind and frequency of use. Using a much denser corpus we show that the relationship between the semantic rating of a verb and its frequency in the language is a relationship that does hold for the *un*-constructions and also holds over the historical period considered here.

So semantics help to explain some of the overall historical trajectories of un-forms yet there is clearly some historical variation left to explain, for example, why particular un-forms of the same “fit” category still show historical variation in frequency *within* their category. One possibility is that importing *close*, for example, into the *un*-construction is blocked because *open* already serves this communicative purpose very well. More than that, we wanted to know (a) whether there is any historical evidence for this idea and (b) whether the skew of the preemptor set also has an effect on the frequency of the *un*-form.

**Figure 3:**
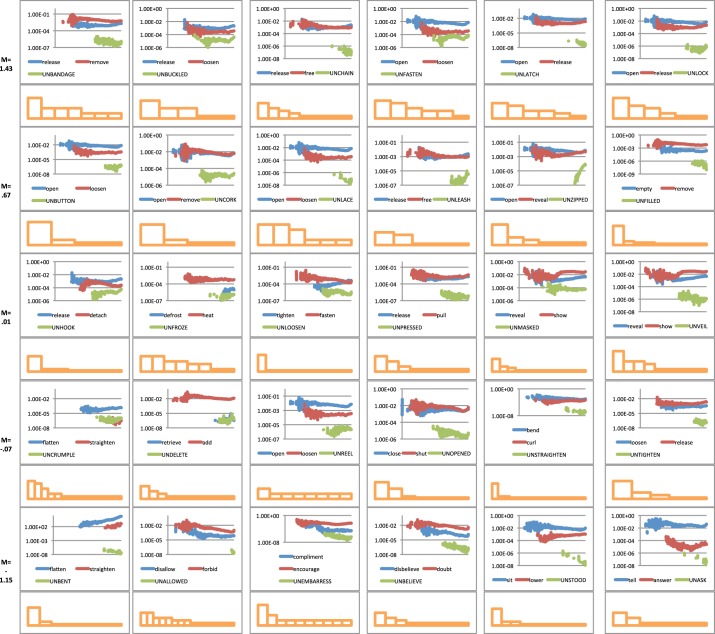
**The historical frequency data of un-forms and their associated top two competitors plus the histogram plots for all preemptors associated with a particular un-verb.** Rows are organized into the same five groups as displayed in Figure 2—from best examples of un-forms at the top through to worst examples at the bottom.^3^

To analyse this possibility six verbs were randomly selected from each semantic group. As this analysis involved many more data points than the previous analysis and much more data processing per analysis, we chose six verbs per category to make the task more manageable. For each *un*-form and its top two semantic competitors we plotted the historical frequency data. As we are also interested in shape of the distribution of the competitors—not just their frequency—we included *all* competitor forms. The skew plots that relate to this appear directly beneath each verbal plot, Figure 3.

In theory, the relationship between un-forms and their pre-emptors could be positive—both forms increase or decrease relative to each other—or negative—as one increases the other decreases. To analyse these possibilities we collapsed the individual verbs displayed in Figure 3 into the semantic cryptotype group used in the previous analysis: best fit, better fit, average fit, poor fit and poorest fit. We took the mean frequency plots for each time point for each verb in their semantic class. We then looked at the relationship between un-form and the averaged over semantic groups by analysing their co-variance, Table 3.

**Table 3:** **Pearson correlation between un-forms and their top two pre-emptors**.

**Semantic rating**	
Best fit	*r*_(485)_ = −0.0007, *p* = 0.88
Better fit	*r*_(485)_ = 0.03, *p* = 0.509
Average fit	*r*_(485)_ = 0.096, *p* = 0.034
Poor fit	*r*_(485)_ = −0.006, *p* = 0.89
Poorest fit	*r*_(485)_ = −0.27, *p* = 0.552

Figure 3 and Table 3 do not provide convincing evidence that un-forms and their top pre-emptors co-vary (either positively or negatively) with only one of the five categories showing a significant correlation and then only explaining ~1% of the variance. If two forms are competing to express a similar concept, one might expect that an increase in the proportion of one form might come at the expense of the other. We investigated this possibility because Michel et al. (2011) found some evidence for this kind of competition with regular/irregular past tense pairs, for example with *chide*/*chode*/*chided* and *sped*/*speeded*/*speed up*. That is, as the frequency of *speed up* increased, the frequency of *sped* decreased. However, *burnt*/*burned* and *snuck*/*sneaked* showed no such relationship. It is this latter kind of null effect we see with the *un*-construction indicating that there is no simple zero-sum relationship between the frequency of competing forms.

**Figure 4:**
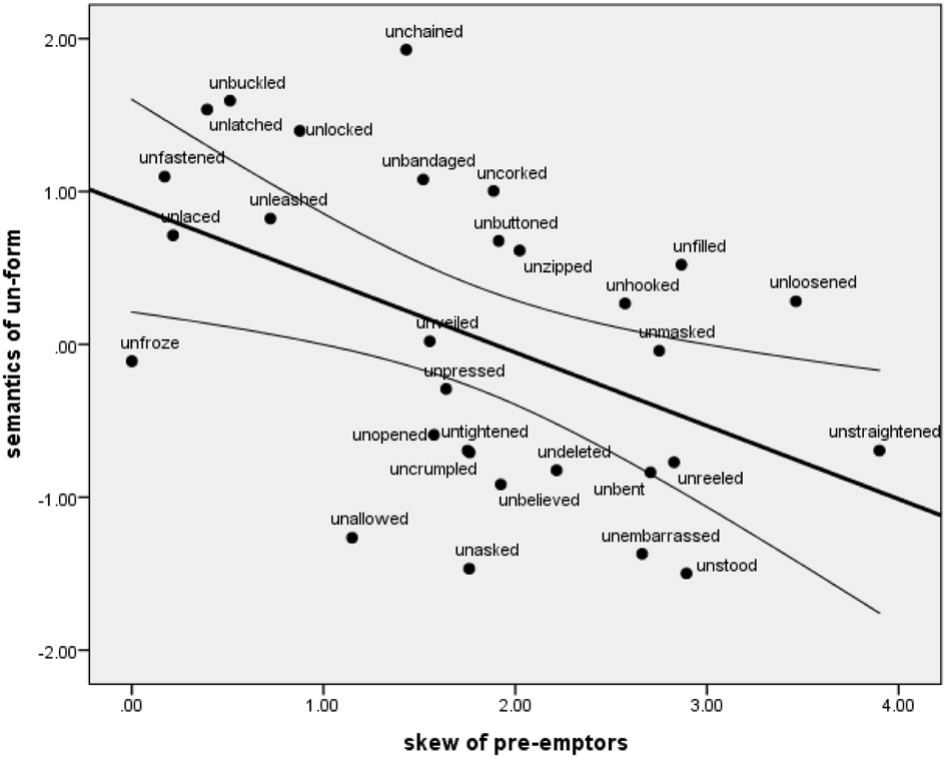
**Correlation between the semantic rating of the verb and the skew of its pre-emptors, *r*_(30)_ = −0.478, *p* < 0.01, line of best fit displayed with 95% confidence intervals**.

What is noticeable from Figure 3 is that as the semantic rating for *un*-forms decrease, the skew of the competitors (displayed in the histogram plots) appears to increase. To examine this further we calculated the skew for each pre-emptor distribution (see Methods) and plotted this against the semantic rating, Figure 4.

We found a significant negative correlation such that poorer examples of the *un*-construction^2^ were more likely to have a one or two highly frequent competitors, whereas for good examples of the un-form were more likely to have a more evenly distributed range of competitors. This is evidence for the skew-preemption hypothesis outlined in the introduction. The cognitive means by which this is suggested to work is as follows. The more the set of relevant pre-emptors is skewed then the more easily an alternative form—that expresses a similar meaning as the *un*-construction—is retrieved from memory because of its higher resting activation level. This then blocks or pre-empts the *un*-form and could provide another explanation as to why these forms have been historically infrequent. Thus, there may be similar processes at work (skew and cognitive anchoring) in restricting *un*-construction generalizations as have been demonstrated in other linguistic and non-linguistic categories (Elio and Anderson, 1984; Avrahami et al., 1997; Gentner et al., 2002; Goldberg et al., 2004; Casenhiser and Goldberg, 2005; Goldberg, 2006, p. 88).

A higher resting activation level is typically determined by frequency of use, with more frequently used words having a lower selection threshold. The top pre-emptors in the skewed set may or may not have a high resting state, thus the selection of the pre-empting form may not be driven by the skew *per se* but the relative frequency of the item. This raises the question of what is the correct set over which to calculate frequency. If one assumes the pre-emptor frequency is calculated over all possible items then the top pre-emptor may or may not have a relative high resting potential. If one assumes however the frequency is calculated over a semantically relevant set of competitors then the top pre-emptor will always have a relatively higher resting activation if it is part of a skewed set. A relevant semantic set must be factored in at some point in processing as if pre-emptors were selected on the basis of raw frequency alone, then highly frequent (the most frequent) items would always be chosen regardless of whether they were semantically acceptable or not. As a reviewer pointed out, in flatter distributions, the best competitor receives more competition for selection from other competitors in their set. So this raises the possibility that it is not that items in a flatter distribution have lower resting activations but that competitors are more comparable in their activation level. In a skewed distribution, all of the poorer competitors are, by definition, poor competitors with the top pre-emptive form making it easier to select.

In the current study the effects of overall frequency and skew of pre-emptors are still somewhat confounded. Recall that Ambridge (2013) found a positive correlation between the raw frequency of the top pre-emptors and the acceptability of their associated un-forms. We find that there is collinear relationship with the skew of the set that these forms happen to part of. This is presumably something which could be experimentally disassociated—for example by controlling for overall frequency of the best competitor and varying skew (and vice versa), and measuring the effect on generalization a novel form.

In conclusion, we found evidence that the extent to which present-day raters consider an *un*-form as a good fit for the semantics of the un-form cryptotype predicts the historical frequency of that form. This indicates this construction's stability for the past 200 years and provides support for the idea that verbal semantic are good predictors of particular usage patterns on a diachronic scale. We did not find strong evidence for direct competition of un-forms with their pre-emptors in the sense that the frequency of one form had a direct effect on the other. However, we did find that the skew of the pre-emptive competitors was inversely correlated with the acceptability of the *un*-form. This suggests that it is not just the raw frequency of the pre-emptive forms that must be taken into account when trying to explain usage patterns but the shape of the distribution as well.

### Conflict of interest statement

The author declares that the research was conducted in the absence of any commercial or financial relationships that could be construed as a potential conflict of interest.

## Footnotes

^1^Year and Gordon (2009) finds a consistent null effect of skewed input on Korean speakers' acquisition of the English ditransitive construction. However when the experiment's full dataset is considered (see Year, 2009), there is evidence that suggests that skewed input gives participants an increased awareness of semantic constraints on the use of the ditransitive early on.

^2^“Construction” is used here to mean a form-meaning pairing, as in the Construction Grammar of Goldberg (1995). It should be noted that neither the analysis nor conclusions necessarily depend on this sense of the word construction; the findings are also consistent with framing the un-form as the more theoretically neutral concept “schema” or as a semantically-constrained lexical rule.

^3^Un-forms are relatively less frequent in the corpus than their preemptors—especially those that are poor fit with the cryptotype. Because of this they are less likely to reach the minimum threshold for inclusion in the corpus. For the Google ngrams corpus any ngram needs to have appeared over 40 times. Is likely that this explains the delayed appearance of un-forms in the x-axis timeline rather than a sudden influx of novel un-forms entering the language at a particular point in time.
